# Muscle Morphology Does Not Solely Determine Knee Flexion Weakness After Anterior Cruciate Ligament Reconstruction with a Semitendinosus Tendon Graft: A Combined Experimental and Computational Modeling Study

**DOI:** 10.1007/s10439-024-03455-7

**Published:** 2024-02-29

**Authors:** Adam Kositsky, Lauri Stenroth, Rod S. Barrett, Rami K. Korhonen, Christopher J. Vertullo, Laura E. Diamond, David J. Saxby

**Affiliations:** 1 Griffith Centre of Biomedical and Rehabilitation Engineering (GCORE), Menzies Health Institute Queensland, Griffith University, Gold Coast, Queensland Australia; 2 Department of Technical Physics, University of Eastern Finland, Kuopio, Finland; 3 Knee Research Australia, Gold Coast, Queensland Australia

**Keywords:** Hamstrings, Harvesting, Orthopedics, Rehabilitation, Strength

## Abstract

The distal semitendinosus tendon is commonly harvested for anterior cruciate ligament reconstruction, inducing substantial morbidity at the knee. The aim of this study was to probe how morphological changes of the semitendinosus muscle after harvest of its distal tendon for anterior cruciate ligament reconstruction affects knee flexion strength and whether the knee flexor synergists can compensate for the knee flexion weakness. Ten participants 8–18 months after anterior cruciate ligament reconstruction with an ipsilateral distal semitendinosus tendon autograft performed isometric knee flexion strength testing (15°, 45°, 60°, and 90°; 0° = knee extension) positioned prone on an isokinetic dynamometer. Morphological parameters extracted from magnetic resonance images were used to inform a musculoskeletal model. Knee flexion moments estimated by the model were then compared with those measured experimentally at each knee angle position. A statistically significant between-leg difference in experimentally-measured maximal isometric strength was found at 60° and 90°, but not 15° or 45°, of knee flexion. The musculoskeletal model matched the between-leg differences observed in experimental knee flexion moments at 15° and 45° but did not well estimate between-leg differences with a more flexed knee, particularly at 90°. Further, the knee flexor synergists could not physiologically compensate for weakness in deep knee flexion. These results suggest additional factors other than knee flexor muscle morphology play a role in knee flexion weakness following anterior cruciate ligament reconstruction with a distal semitendinosus tendon graft and thus more work at neural and microscopic levels is required for informing treatment and rehabilitation in this demographic.

**Supplementary Information:**

The online version contains supplementary material available at 10.1007/s10439-024-03455-7.

## Introduction

Anterior cruciate ligament (ACL) ruptures are increasingly common worldwide, particularly in young people [76, 92, 98], and impose substantial socioeconomic burden [44, 58]. Reconstruction of the ACL (ACLR) is commonly performed using an autologous ipsilateral distal semitendinosus tendon autograft [22]. An ACLR generally enables individuals to return to physical activity, albeit sometimes well below pre-injury levels of activity [14, 78]. Following semitendinosus tendon ACLR, there are long-term morphological alterations to the semitendinosus muscle, such as reductions in muscle volume, cross-sectional area, and length [10, 32, 39, 45, 50, 52, 55, 56, 74, 94], and substantial deficits in knee flexion strength, particularly with the knee in more flexed positions [2, 16, 39, 43, 50, 55, 56, 69, 79, 83]. Crucially, these morphological and strength changes occur even when the semitendinosus tendon regenerates and reattaches near its original insertion point [62, 75]. Atrophy of semitendinosus and knee flexion weakness are likely linked and may both contribute to lower physical activity levels and decreased physical performance post- compared to pre-ACLR. However, whether semitendinosus atrophy alone is the causal factor for knee flexor weakness post-ACLR is not clear.

In those with tendon regeneration, altered semitendinosus muscle morphology post-ACLR can affect knee flexion strength in two ways. Atrophy of semitendinosus radially (i.e., reduction in muscle cross-sectional area) will contribute to knee flexion weakness as the maximal force-producing capacity of the muscle is diminished due to fewer sarcomeres in parallel. Longitudinal atrophy (i.e., shortening of resting semitendinosus muscle length) occurs from a reduction in the number and/or length of serial sarcomeres. These changes at the sarcomere level following the disruption of the muscle-tendon-bone connection affect muscle operating range [90], influencing the post-ACLR semitendinosus force-joint angle relationship and leading to joint angle-dependent weakness. The extent to which radial and longitudinal atrophy individually contribute to, and whether they together wholly explain, post-ACLR knee flexion weakness is unclear as studies examining links between post-ACLR semitendinosus morphology and knee flexion strength are equivocal [10, 32, 39, 50, 52, 56]. The inconsistency across studies may stem from some participant samples being confounded by including those without semitendinosus tendon regeneration, but also from potential adaptations of synergist knee flexor muscles to varying extents. These synergist muscles might adapt to the loss of semitendinosus function post-ACLR via compensatory hypertrophy [32, 45, 74] and/or by increasing activation levels during certain tasks [45, 82]. However, synergist compensations are not always found [43, 56, 81] and are potentially more likely to occur when the distal semitendinosus tendon does not regenerate after harvesting [17]. More fundamentally, it is unclear whether these synergist muscles have the capacity to compensate for impaired, or potential total loss of (in the event of failed tendon regeneration), semitendinosus function.

Most studies that have examined the relationship between hamstring morphology and knee flexion strength post-ACLR have been cross-sectional in design and used correlation analyses [10, 32, 39, 50, 52, 56]. However, cross-sectional correlations are limited by the substantial measurement error associated with laboratory strength testing [21, 85], and, more importantly, any reported correlation does not infer a causative relationship. Studies have also made inferences based on subgroups stratified by semitendinosus morphology in the ACLR compared with the contralateral leg [55, 56], but such approach cannot separate nor quantify the independent effects of radial and longitudinal atrophy. A complimentary method to experimental measurement is musculoskeletal modeling and simulation, which, although subject to its own limitations, is sensitive to changes in relevant morphology-based inputs (e.g., [7, 72]), enabling interrogation of the causative relationship between muscle morphological factors and knee flexion strength post-ACLR. To our best knowledge, no study has used computational methods to investigate the effect of muscle morphology on knee flexor weakness after harvesting the distal semitendinosus tendon for ACLR. Therefore, the main purpose of this study was to combine experimental and computational approaches to determine, during maximal isometric knee flexion contractions in individuals post-ACLR with a semitendinosus tendon autograft, the effects of semitendinosus muscle atrophy on knee flexion weakness. Secondly, we also assessed whether the knee flexor synergists can compensate for the reduced semitendinosus contribution to knee flexion strength.

## Materials and Methods

### Participants

Ten individuals who were 8–18 months post-ACLR at the time of experimental testing and imaging participated in this study (Table 1). For each participant, ACLR was performed using a quadrupled distal semitendinosus tendon autograft, with the gracilis tendon left intact [35]. All experimental data were acquired from both the ACLR and contralateral legs in identical fashion, with imaging data from these participants having been previously published [33, 35]. The ACL injury and subsequent ipsilateral ACLR (semitendinosus tendon autograft) occurred on the non-dominant side for 90% of participants, where leg dominance was defined as the leg most commonly used to kick a ball. Participants were requested to refrain from strenuous exercise commencing 24 h prior to any involvement in the study. The study was carried out as per the Declaration of Helsinki and received ethical approval from the Griffith University Human Research Ethics Committee (2018/839). Written informed consent was obtained prior to any participation in the study.

**Table 1:** Descriptive characteristics (mean ± standard deviation) for all participants and subgroups stratified by distal semitendinosus tendon regeneration in the anterior cruciate ligament reconstructed leg.

	Total (*n* = 10)	Regenerated (*n* = 7)	Non-regenerated (*n* = 3)
Sex	4M; 6F	4M; 3F	0M; 3F
Age (years)	27.2 ± 4.9	27.8 ± 5.7	25.8 ± 2.7
Height (m)	1.72 ± 0.10	1.74 ± 0.11	1.67 ± 0.02
Mass (kg)	72.6 ± 13.4	75.8 ± 14.6	65.2 ± 7.7
Time post-surgery (months)	13.9 ± 3.4	14.1 ± 3.6	13.5 ± 3.4

### Maximal Isometric Knee Flexion Moment

Participants performed a warm-up consisting of 5 min on a cycle ergometer at a self-selected pace followed by a series of unilateral submaximal isometric knee flexion contractions and passive knee extensions. Isometric knee flexion maximal voluntary contractions (MVC) were performed on an isokinetic dynamometer (System 4 Pro, Biodex, Shirley, NY, USA) at four knee joint angles: 15°, 45°, 60°, and 90° (0° = extension). The order of legs and the order of joint angles were randomized. Torque and joint angle were recorded at 2000 Hz with Vicon Nexus (Version 2.9.1, Vicon, Oxford Metrics Group, UK). Note that although the isokinetic dynamometer records torque, herein it is referred to as moment for consistency across methods. Participants were positioned prone (hip = 0°) on a plinth with supportive straps fastened around their waist. An ankle cast (AirSelect Short, Aircast, Vista, CA, USA) was used to standardize and maintain neutral ankle angle to eliminate the effects of ankle angle on isometric knee flexion moment [59]. The dynamometer lever arm was attached to the top of the ankle cast and the knee joint center was aligned to the center of rotation of the dynamometer. The knee joint angle was set using the calibrated dynamometer and confirmed with a hand-held goniometer. At each knee joint angle, a minimum of three trials were performed with 1 min rest between trials. Further trials were performed if the third, or subsequent trials, were at least 5% greater than the maximum of any previous trial [21]. A 2 min break was given between knee joint angle changes and at least 5 min rest between legs.

Custom-written scripts in MATLAB (version R2018b, MathWorks, Natick, MA, USA) were used to analyze each trial. Signals were first low-pass filtered at 20 Hz with a zero-lag, fourth-order Butterworth filter [67]. For each trial, moment values were calculated as the maximum 500 ms moving average from which average baseline moment over a 500 ms period was subtracted to account for the weight of the limb and remove the contribution of passive elements. The maximum value of all trials was recorded as MVC for a given joint angle. The relative difference between legs was calculated by subtracting the contralateral leg moment from the ACLR leg moment and then dividing by the contralateral leg moment.

### Musculoskeletal Model Representing the Contralateral Legs

A computer simulation using OpenSim (v4.1, SimTK, Stanford, CA, USA) was performed using a customized version of the Lai et al. [38] model. The model was not subject-specific but rather a single model representing the average data from the contralateral (intact healthy) leg of the seven participants who had distal semitendinosus tendon regeneration on their ACLR leg. The contralateral leg was considered to represent the properties of the ACLR leg before the injury and reconstruction. Data from participants without distal semitendinosus tendon regeneration (i.e., a tendinous structure below the knee joint was not visible from two-dimensional proton density and three-dimensional T_1_ Dixon magnetic resonance imaging (MRI), as determined by an experienced examiner [35]) were not included in the model and excluded from simulations as the loss of their distal semitendinosus tendon insertion point on the tibia means force generated by semitendinosus in these individuals can only be transferred through epimuscular pathways. Although apparently evident in cats [91], it is unknown to what extent myofascial force transmission occurs among the human hamstrings and thus was not incorporated or represented in the musculoskeletal model.

The model and all simulations were controlled by accessing the OpenSim application programming interface (API) using custom-made MATLAB scripts. In the model, the hip was locked at 0° (neutral) to reflect the prone position used for experimental testing, with the ankle also locked in neutral at 0°. The four hamstring muscles, four quadriceps muscles, gracilis, and sartorius were represented using Hill-type muscle models [84]. The gastrocnemii were not included in the model (see Limitations). The gross dimensions, mass, and inertia of the pelvis, femur, patella, and tibia in the model were scaled based on the distance between the greater trochanter and lateral femoral condyle on the contralateral leg for these seven participants (mean = 41.3 cm), as measured with a flexible tape measure. We consider this scaling method valid given the high correlation between segment lengths in humans [30].

For all muscles other than semitendinosus, the optimal fiber (ℓf_o_) and tendon slack (ℓT_s_) lengths were optimized following model scaling to maintain force–length curves for each muscle [48]. Muscle volumes were estimated using regression equations [25] incorporating the average mass and height of the participants in the modeled group (Table 1). Then, the maximal isometric force (F_max_) for these muscles was adjusted:

1 $${{\text{F}}}_{max}= {\sigma }_{m}\times (\frac{{\text{Volume}}}{\ell{{\text{f}}}_{o}})$$

where *σ*_m_ is muscle specific tension, set to 60 N/cm^2^ as per the default in the template model adopted for this study [38, 65]. The rationale for this *σ*_m_ value is described in more detail elsewhere [65] but is nonetheless within the range reported previously for the human hamstring muscle group *in vivo* [42].

As studies suggest proximal and distal semitendinosus compartments function together as a single unit [35, 36], semitendinosus was modeled as one segment. Using published MRI data from these seven participants (Table 2 of Kositsky et al. [35]), we adjusted semitendinosus based on their contralateral leg. First, ℓf_o_ of semitendinosus was recalculated from an established equation [23, 89]:

2 $$\ell{{\text{f}}}_{o}=\ell{{\text{f}}}_{anat}\times (\frac{2.7\mathrm{\mu m}}{\ell{\text{sarc}}})$$

where ℓf_anat_ and ℓsarc are fiber and sarcomere length, respectively, in the anatomical position. Generally, semitendinosus fascicles run from tendon origin/insertion to the tendinous inscription that divides the muscle into proximal and distal compartments, and there is no evidence for intrafascicularly terminating semitendinosus fibers in humans [95]. Therefore, we took the average of the mean proximal (20.5 cm) and distal (24.5 cm) compartment longitudinal lengths from these participants [35] for ℓf_anat_ (22.5 cm). Although this method may overestimate fiber length, as the oblique nature of the tendinous inscription and muscle-tendon junctions means fibers do not span the entire longitudinal component of a compartment, using compartment lengths allowed for the best and most realistic personalization possible for our data, and the ℓf_anat_ we implemented is comparable with the resulting length when summing the two serial fiber lengths [23, 89]. For determining semitendinosus ℓf_o_, a value of 2.89 μm was used for ℓsarc, obtained from cadaveric data [89] acquired with the hip and knee in approximately the same position as during MRI scans in our experimental setup (i.e., the anatomical position).

**Table 2:** Means and standard deviations of absolute moment (Nm) for knee flexion maximal voluntary isometric contractions at four knee joint angles for contralateral and anterior cruciate ligament reconstructed (ACLR) legs. Between-leg differences were calculated relative to the contralateral leg for each participant and are presented as means and 95% confidence intervals of all individual calculations (rather than based on mean absolute values). Repeated measures ANOVAs were performed on the absolute values for the entire sample and for the tendon regenerated subgroup but not for the non-regenerated subgroup due to the sample size. Refer to the main text for the main effects and interactions. Note the lower absolute values in the non-regenerated subgroup, consistent with smaller body dimensions presented in Table 1.

	Total (*n* = 10)			Regenerated (*n* = 7)			Non-regenerated (*n* = 3)		
	Contralateral	ACLR	Difference	Contralateral	ACLR	Difference	Contralateral	ACLR	Difference
15°	87.4 ± 35.8	83.2 ± 38.5	− 5.8 ± 9.5%	102.2 ± 32.2	99.8 ± 33.7	− 2.0 ± 10.9%	53.0 ± 11.3	44.3 ± 5.9	− 14.7 ± 36.6%
45°	77.0 ± 30.2	68.9 ± 25.2	− 9.7 ± 9.8%	88.0 ± 29.6	80.8 ± 19.5	− 5.3 ± 11.9%	51.5 ± 8.3	41.2 ± 8.1	− 19.9 ± 27.6%
60°	71.6 ± 28.8	56.2 ± 23.1*	− 21.7 ± 7.6%	83.2 ± 26.5	66.6 ± 19.1*	− 18.7 ± 10.5%	44.5 ± 6.7	31.9 ± 6.8	− 28.6 ± 11.3%
90°	55.6 ± 19.8	37.4 ± 13.7**	− 32.4 ± 6.2%	63.5 ± 18.3	42.9 ± 12.7*	− 32.1 ± 7.9%	37.3 ± 5.6	24.5 ± 1.4	− 33.2 ± 26.4%

*(*p* < 0.01) and **(*p* < 0.001) significantly different from the contralateral leg for statistical comparisons within subgroups (note no between-subgroup comparisons were made)

Subsequently, F_max_ for semitendinosus was calculated using maximal anatomical cross-sectional area (ACSA_max_):

3 $${{\text{F}}}_{max}= {\sigma }_{m}\times {\text{ACSA}}_{max}$$

where *σ*_m_ was assumed to be unaffected by ACLR and ACSA_max_ for the tendon regenerated subgroup was 11.8 cm^2^ [35]. We chose to use ACSA_max_ for determining F_max_ rather than volume (Eq. 1) for two reasons. First, as semitendinosus is parallel-fibered, ACSA_max_ is a good proxy of physiological cross-sectional area [23]. Second, using ACSA_max_ allowed us to separate the effects of radial and longitudinal morphological changes. Eq. 1 incorporates both muscle volume and ℓf_o_ when calculating F_max_, precluding assessment of the individual contributions of radial and longitudinal factors. After F_max_ was determined, ℓT_s_ for semitendinosus was adjusted by finding the value providing the model with a fiber length in the anatomical position that matched ℓf_anat_ (22.5 cm) with an activation of 0.01 [11, 65].

**Fig. 1:**
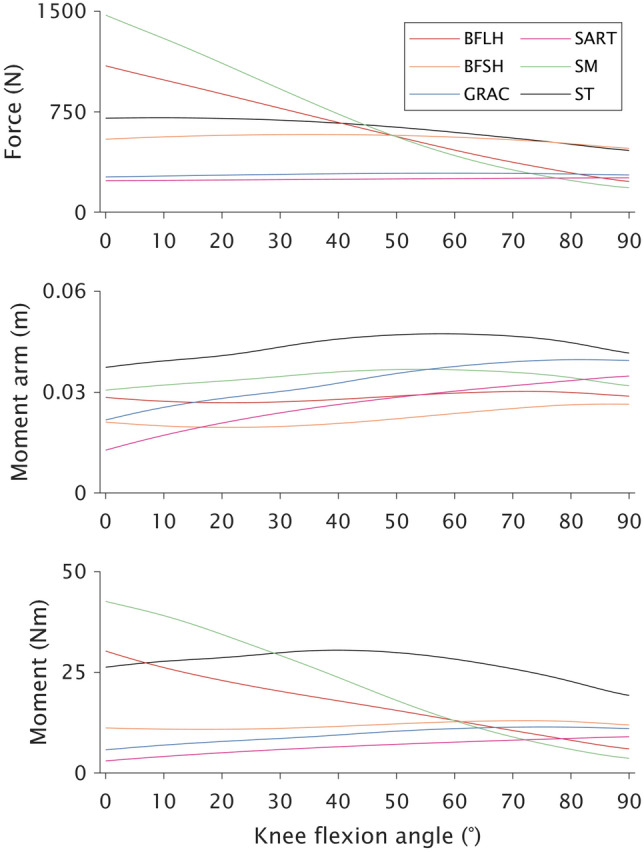
Baseline model outputs of active fiber force (top), moment arm (middle), and knee flexion moment (bottom) produced by each muscle for the long (BFLH; red) and short (BFSH; orange) heads of the biceps femoris, gracilis (GRAC; blue), sartorius (SART; magenta), semimembranosus (SM; green), and semitendinosus (ST; black) muscles from full knee extension (0°) to 90° of knee flexion with the hip neutral (0°). Activation was set to maximal for all muscles across the entire range of motion. Note the high contribution of semitendinosus to the overall knee flexion moment at high knee flexion angles

To better represent the *in vivo* force–length relationship of human skeletal muscle during MVC [41], the value for the active Gaussian shape factor was set to 0.20 for all muscles (Supplementary Figure 1). As semitendinosus, gracilis, and sartorius are parallel-fibered, we assumed no pennation angle in these muscles [43]. All tendons in the model were considered elastic with strain at maximal isometric force set to 4.9% [65]. The active fiber force, moment arm, and knee flexion-moment curves for each knee flexor muscle under maximal activation are presented in Figure 1.

**Fig. 2:**
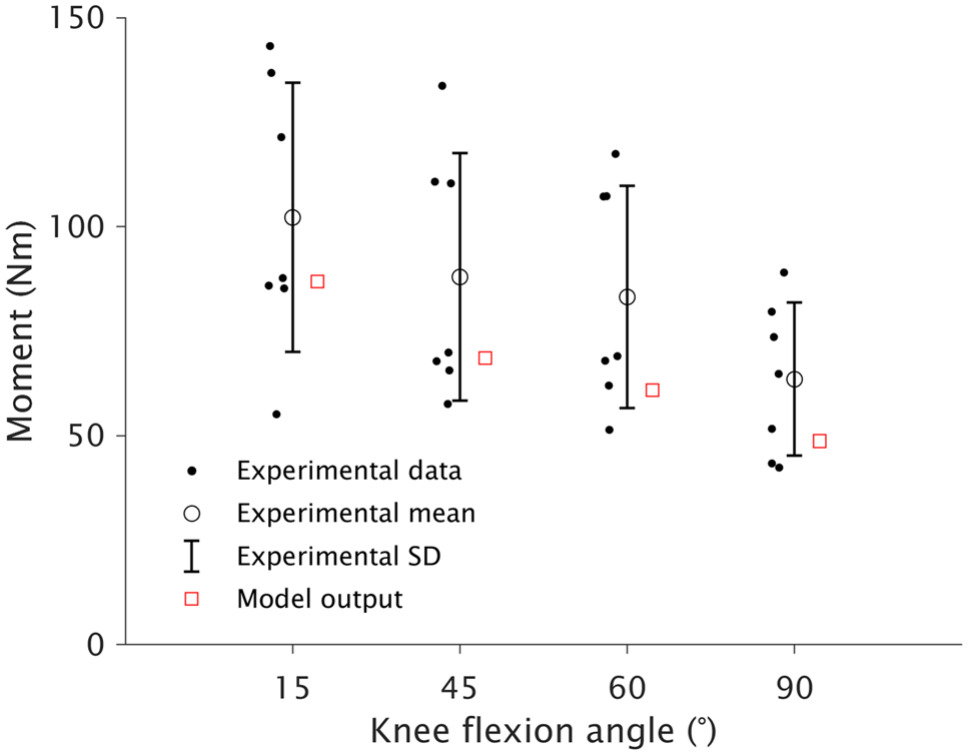
Means and standard deviations (SD) of knee flexion moments produced by the contralateral leg of participants in the tendon regeneration subgroup (black circles; *n* = 7) at four knee joint angles compared to the output of the representative baseline musculoskeletal model (red squares). Closed black circles represent individual datapoints. All model outputs were within one SD of the experimentally produced moment

For simulations, all knee flexor muscles were maximally activated. As the resultant knee flexion moment measured during experimental testing is reduced by the knee extension moment generated by co-activation of the quadriceps, activation in the model for each quadricep muscle was set at 0.075 [3, 37]. At each knee joint angle (15°, 45°, 60°, and 90°), the active knee flexion moment (agonist) less the active knee extension moment generated by the quadriceps (antagonist) was calculated and considered to be the baseline knee flexion moment (resultant) produced by the contralateral leg (Figure 2). Subsequent simulations after adjusting muscle parameters (see Adjusted Model and Simulations for the ACLR Leg) are compared against this value, deemed to be the reference value for each joint angle.

### Adjusted Model and Simulations for the ACLR Leg

**Fig. 3:**
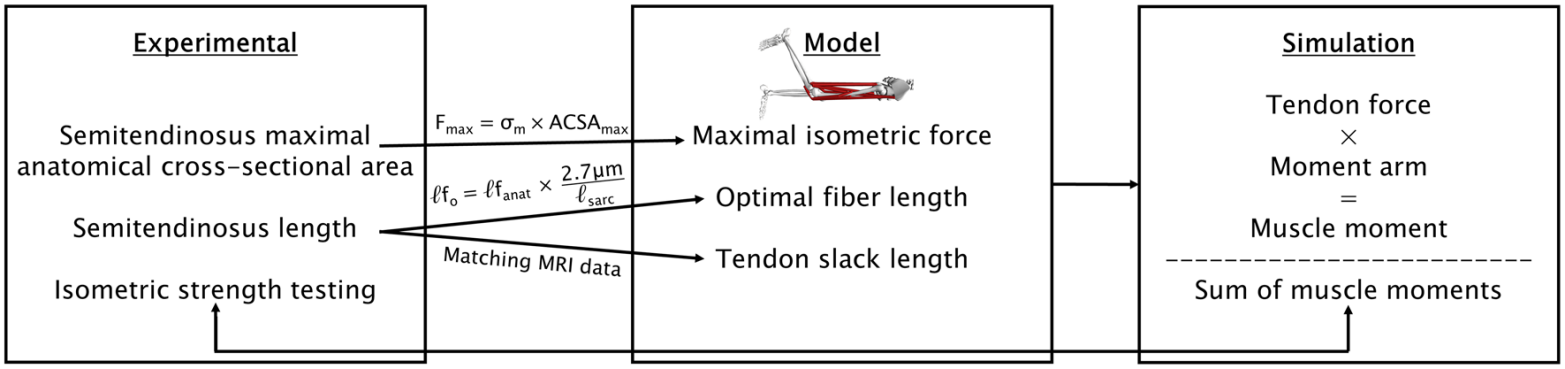
Summary of the workflow for simulations investigating the effects of semitendinosus morphological changes on knee flexor strength. Semitendinosus maximal anatomical cross-sectional area and length obtained via magnetic resonance imaging (MRI) were used to adjust key muscle model parameters. The simulated resultant knee flexion moments (sum of all agonists minus sum of all antagonists) were compared with experimental strength data obtained with an isokinetic dynamometer. *ACSA*_*max*_* = *maximal anatomical cross-sectional area; *F*_*max*_* = *maximal isometric force; *ℓf*_*anat*_* = *fiber length in the anatomical position; *ℓf*_*o*_* = *optimal fiber length; *ℓ*_*sarc*_* = *sarcomere length in the anatomical position; *σ*_*max*_* = *specific tension

Simulations assessing the effects of semitendinosus morphogical changes in the ACLR leg on knee flexion moment are summarized in Fig. 3 and were informed by previously published morphological data from these participants [35], presented below in percentages. The relative between-leg differences in semitendinosus muscle ACSA_max_ and length were used to adjust F_max_ and ℓf_o_, respectively, in 1% intervals between the upper and lower bounds of one standard deviation of the mean between-leg differences. For effects of radial morphological changes, semitendinosus F_max_ was adjusted by changing ACSA_max_ from + 8% to − 22% in Eq. 3. Note the relatively large standard deviation resulting in positive values was due to two participants having a larger semitendinosus ACSA_max_ on their ACLR compared to contralateral leg. As the chronic adaptation of semitendinosus sarcomeres post-ACLR remains unknown, the difference in semitendinosus length was attributed to either a loss of sarcomeres in series or a reduction in ℓsarc in the anatomical position to account for both possible adaptations independently. To decrease serial sarcomere number, ℓf_anat_ was reduced by 6–17%, and for each iteration ℓf_o_ was determined by Eq. 2 with ℓsarc in the resting anatomical position unchanged. For shortening sarcomeres, Eq. 2 was again used, but ℓf_anat_ and ℓsarc were both adjusted from − 6% to − 17% (by the same percentage for each iteration). For all iterations involving sarcomere-level modifications, ℓT_s_ was re-determined in the same manner as for the contralateral leg based on the “new” ℓf_anat_ for each simulation. Each individual morphological factor was simulated in isolation and then the changes in ACSA_max_ were combined with either a change in ℓsarc number or length.

The simulations were performed with the following considerations. Muscle activation levels were unchanged from baseline simulations as we focused on the effect of morphological adaptations. We also did not adjust F_max_ of knee flexor synergists for these simulations as (i) the presence of compensatory hypertrophy is inconsistent across studies [32, 43, 45, 56, 74, 81], (ii) additional simulations were performed increasing F_max_ of these muscles (see next paragraph), and (iii) these main simulations focused on determining the effects of semitendinosus atrophy alone. However, when calculating the knee extension co-contraction moment, F_max_ of each quadricep muscle was reduced by 10% to account for known and consistently reported quadriceps atrophy in individuals with semitendinosus ACLR [32, 74, 94], although co-activation level was unchanged [54]. For each simulated condition, the adjusted active extension moment (antagonist) was subtracted from the active flexion moment (agonist), and the resultant knee joint moment for each iteration compared against the reference value from the baseline model (i.e., contralateral leg) at that joint angle. It is crucial to note that due to the quadriceps atrophy-induced reduction in the knee extension co-contraction moment, the resultant knee flexion moment can be greater than the baseline model even when accounting for ACLR-related morphological changes to semitendinosus.

To assess the potential for the synergist knee flexor muscles to compensate for the affected semitendinosus, additional simulations were performed. We first artificially impaired semitendinosus in the model such that, at a given joint angle, the resultant modeled knee flexion moment relative to the baseline model (i.e., “between-leg” model difference) matched the between-leg difference in experimental moment. Then, F_max_ of the semitendinosus synergists were all increased in 1% intervals up to 20% to mimic potential compensatory hypertrophy. The F_max_ increases were performed for all synergists simultaneously and not independently for any single muscle. For each iteration of increased synergist F_max_, the co-contraction extension moment for the ACLR leg (adjusted with the 10% quadriceps atrophy) was subtracted from the active flexion moment, with model outputs compared to those from the baseline model.

### Statistical Analyses

Knee flexion moment values for both legs were normally distributed at all joint angles (Shapiro–Wilk test; *p* > 0.05). To assess the effects of leg and knee angle on knee flexor moment, two-way repeated measures ANOVAs were performed on the entire sample (*n* = 10) and repeated including only those participants with distal semitendinosus tendon regeneration (*n* = 7). When significant main effects or interactions between leg and knee angle were detected, Bonferroni corrections were applied to pairwise comparisons by multiplying the raw *p*-value by the respective number of multiple comparisons (leg = 2, angle = 4, interaction = 8). Statistical analyses were performed using SPSS (v27, IBM Corp., Armonk, NY, USA), and statistical significance was accepted for *p* < 0.05.

To assess whether semitendinosus muscle morphology post-ACLR could fully result in the decrease in knee flexor moment observed empirically, simulated maximal moment production (relative to the reference model value) was compared to the mean ± 95% confidence intervals of the experimental between-leg differences in moment. If the model estimates for a given simulation condition overlapped the 95% confidence intervals (Student’s *t*–distribution method) from the experimental results, we interpreted this as that condition having the capacity to fully induce the resulting knee flexion weakness.

To assess whether knee flexor synergists could potentially compensate for impaired semitendinosus contribution to knee flexion moment, simulated maximal moment production was compared to the baseline reference model. Synergists were considered to compensate for the knee flexion weakness if the simulated flexion moment reached or surpassed the baseline model (considered 0% moment difference) at any step interval of F_max_ increase.

## Results

### Experimental Findings

Seven out of ten participants had regeneration of their semitendinosus tendon following ACLR. For the entire sample (*n* = 10), a significant interaction between leg and angle (*p* = 0.006) for MVC revealed the ACLR leg to be significantly weaker than the contralateral leg at 60° (*p* = 0.001) and 90° (*p* < 0.001), but not at 15° (*p* = 0.305) or 45° (p = 0.061) (Table 2). Likewise for the regenerated tendon subgroup (*n* = 7), there was a significant interaction between leg and angle (*p* = 0.010) due to the ACLR leg producing significantly less moment than the contralateral leg at 60° (*p* = 0.008) and 90° (*p* = 0.001), but not at 15° (*p* = 0.668) or 45° (*p* = 0.229).

### Computational Findings

**Fig. 4:**
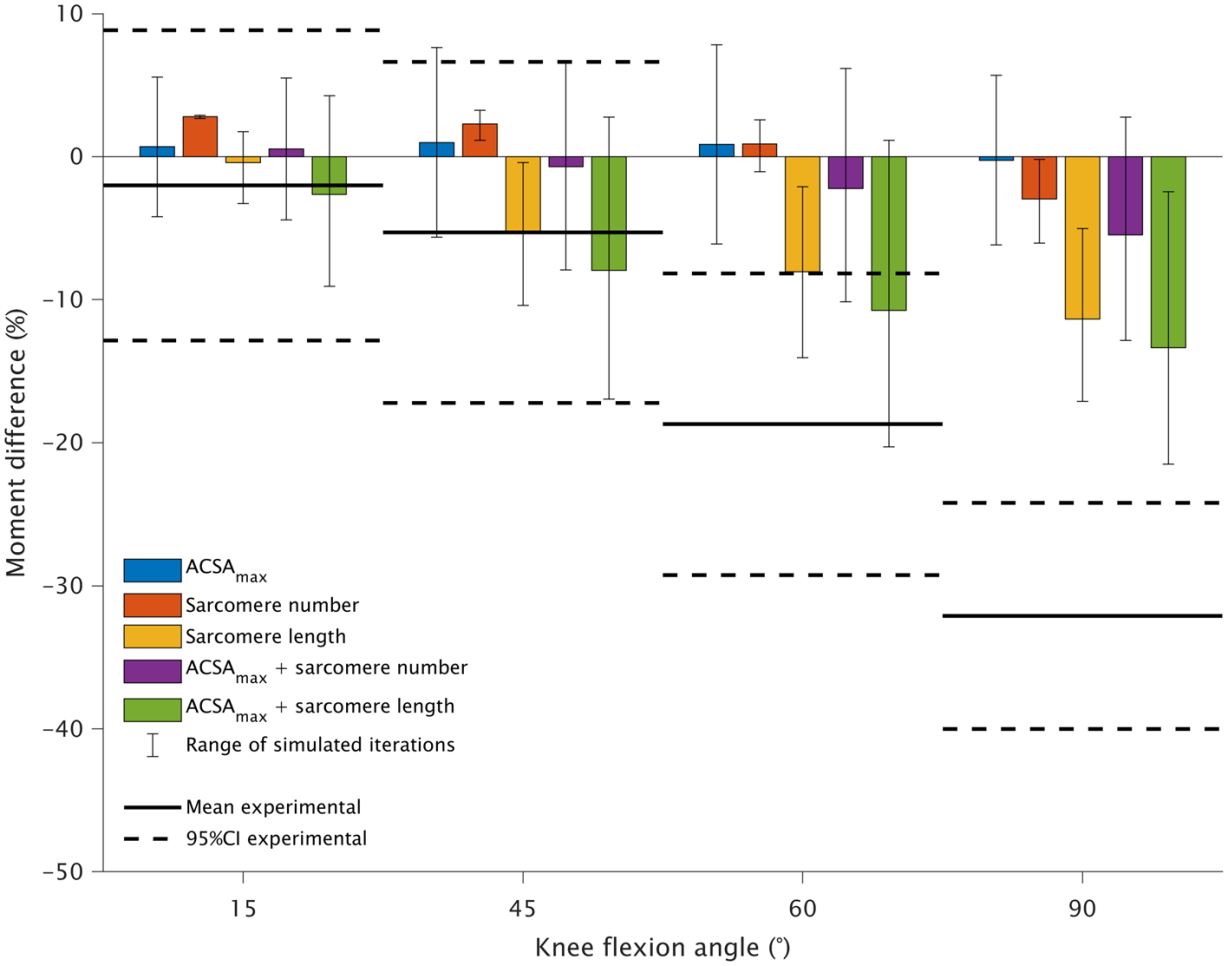
Means (horizontal solid black line) and 95% confidence intervals (95%CI; horizontal broken black lines) of the between-leg difference in experimentally produced knee flexion moment for participants with tendon regeneration (*n* = 7) at four knee joint angles compared with model outputs relative to the baseline model after simulating morphological alterations of reduced maximal anatomical cross-sectional area (ACSA_max_; blue) and sarcomere number (red) and length (orange). Reduced ACSA_max_ was also combined with fewer (purple) and shorter (green) sarcomeres. Model values are presented as medians, with the error bars representing the minimal to maximal outputs from the range of iterations used for simulations

At each joint angle, the simulated knee flexion moment of the representative baseline model fell within one standard deviation of the experimentally-measured knee flexion moment for participants with tendon regeneration (Fig. 2). At 60°, only simulations incorporating changes at the sarcomere level could result in similar knee flexion moments seen experimentally (Fig. 4). However, no simulation resulted in the reduction in knee flexion moment at 90°.

**Fig. 5:**
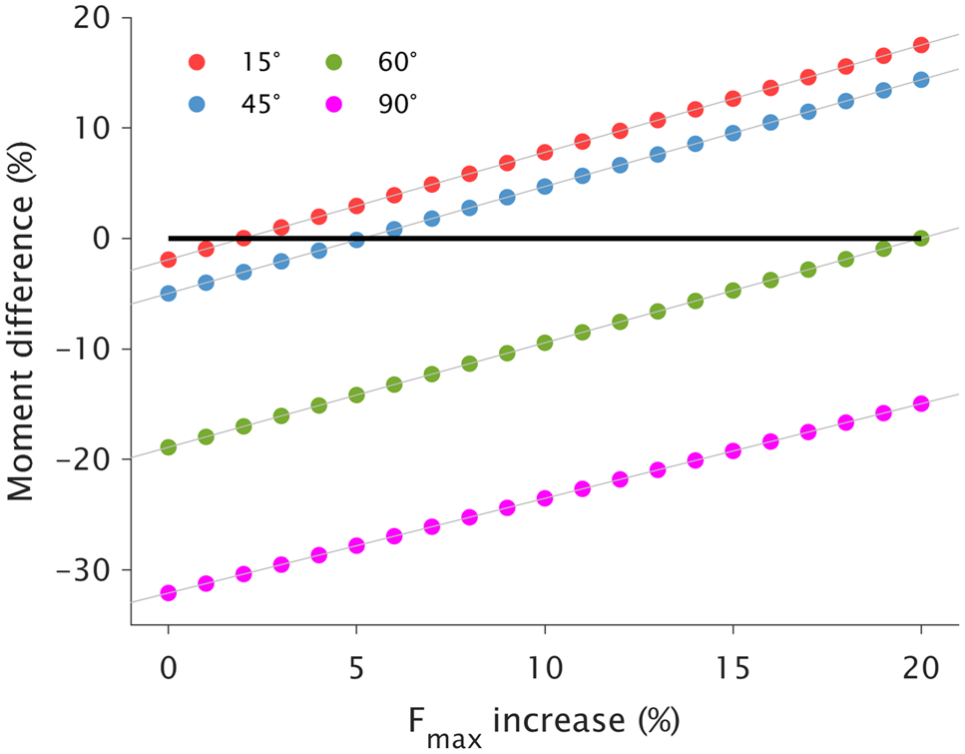
The effects of increasing maximal force producing capacity (F_max_) of knee flexor synergist muscles in one percent step intervals up to 20% at 15° (red), 45° (blue), 60° (green), and 90° (magenta) of knee flexion with the hip neutral. A 0% “increase” in F_max_ (black horizontal line) represents the artificially-impaired semitendinosus model matching the experimental between-leg difference in moment. The synergist-simulated model was considered to have compensated for the impaired semitendinosus if any simulation iteration reached or surpassed 0% moment difference

A 2% increase in F_max_ for the synergists resulted in moment matching the baseline model at 15° of knee flexion (17.5% higher moment with 20% F_max_ increase), while an increase of ~ 5% F_max_ was required at 45° (14.4% higher moment with 20% F_max_ increase) (Fig. 5). At 60°, only a 20% increase in synergist F_max_ was able to match the baseline model moment, but knee flexion was still −14.9% weaker at 90° with this maximum imposed F_max_ increase.

## Discussion

This study combined experimental and computational approaches to elucidate the role of muscle morphology on knee flexion strength in individuals post-ACLR with an ipsilateral distal semitendinosus tendon autograft. The between-leg differences in experimental maximum isometric knee flexion strength following ACLR (i.e., weakness in the ACLR limb) were more pronounced as knee flexion angle increased, but computational musculoskeletal modeling indicated knee flexion weakness was not solely explained by semitendinosus muscle atrophy, particularly at 90°. Modeling also demonstrated that changes at sarcomere level, especially shortening of sarcomeres, negatively influenced knee flexion moment more than did reduced semitendinosus radial size. Further, knee flexor synergists could not compensate for the reduced contribution of semitendinosus to maximum isometric knee flexion moment at high angles of knee flexion. These results suggest other factors, in addition to muscle morphology, contribute to knee flexion weakness post-ACLR and provide important context for clinical assessments after ACLR with a semitendinosus tendon graft.

### Knee Flexion Strength and the Effects of Semitendinosus Atrophy

Consistent with the literature, maximum isometric knee flexion moment was substantially lower with the knee in high angles of knee flexion (Table 2). Specifically, the between-leg difference of ~ 32% at 90° for both the whole sample and the regenerated tendon subgroup are well within the literature bounds of a deficit of ~ − 20% to ~ − 45% at this joint angle [43, 50, 55, 56, 69, 83], and the ~ 20% difference at 60° corresponds well with literature values for both 60° (~ 16-20%; [56, 69]) and 70° (~ 20%; [50, 83]) of knee flexion. Although unable to statistically subgroup due to sample size, the non-regenerated tendon subgroup (*n* = 3) appear weaker than those with tendon regeneration at all angles except 90° (Table 2). Thus, our choice of using data for computations from only the regenerated tendon subgroup was warranted and overall the experimental data are sufficiently representative to serve as a valid comparator for our modeling results.

Using this musculoskeletal modeling approach, we found radial semitendinosus muscle atrophy to, on average, have minimal effect on knee flexion moment in all tested positions (Fig. 4). Note the increases in knee flexion moment evident in some simulated conditions are due to quadriceps atrophy (see Adjusted Model and Simulations for the ACLR Leg and Potential Synergist Compensation and Clinical Implications subsections for more detail). Although unable to fully explain the weakness observed at 90°, simulations including shortening of semitendinosus sarcomeres resulted in greater loss of moment than simulating only radial atrophy. It is unknown if semitendinosus sarcomeres are shorter or are lost after distal semitendinosus tendon harvesting, although both phenomena have occurred in torn human shoulder muscles [19, 87]. It may be unlikely that semitendinosus sarcomeres become shorter in the anatomical position post-ACLR. Supplementary simulations with the hip in 90° of flexion suggest this phenomenon would make individuals substantially stronger at very long muscle lengths (Supplementary Figure 2). Assuming the model is accurate for such conditions (we did not obtain experimental strength data with the hip flexed), such increase in strength does not match literature reports of substantial knee flexion weakness in a joint configuration similar to this supplementary simulation [73]. Nonetheless, both a reduction in sarcomere number or length (or a combination of the two) will result in less force production compared to the normal state of the muscle at short muscle lengths (i.e., high angles of knee flexion) due to alterations in the semitendinosus force–joint angle relationship. Both phenomena would also affect the angle of peak knee flexion moment, shown by other studies to occur at more extended angles (i.e., longer relative muscle length) after the distal semitendinosus tendon is harvested for ACLR [1, 60]. Future studies using microendoscopy and ultrasonography to assess sarcomeric adaptations [64] may be beneficial in elucidating the mechanism leading to the change in semitendinosus operating range.

In the present study, neither radial nor longitudinal atrophy could fully account for the weakness at 90° of knee flexion post-ACLR. The finding that muscle morphology only partially contributes to knee flexion weakness is consistent with Meyer [46], who reported changes in muscle architecture do not solely explain muscle force loss after tenotomy of the mouse rotator cuff. Experiments on animals also found the loss of maximal tetanic force after tenotomy exceeded that which could be predicted from the loss in muscle weight [53]. At 90° of knee flexion, the contribution of semitendinosus post-ACLR appears to be minimal given apparently similar knee flexion moments in those with and without a regenerated distal semitendinosus tendon (Table 2), despite the latter having no distal insertion and greater between-leg morphological differences [35]. Therefore, in addition to changes in the length and/or number of sarcomeres, there may be disorganization of structures within and between sarcomeres [8, 29, 86], leading to impaired force generation. Potential harvesting-induced reductions in dystrophin content (shown to occur after tenotomy [9]) could also negatively affect force transmission pathways [28, 66]. Fatty infiltration to semitendinosus post-ACLR [74, 88] may also lead to weakness [4], as may the fasciotomy performed during the tendon harvesting procedure [18, 68]. Further, greater compliance of the regenerated compared to the native semitendinosus tendon [77] would result in greater shortening of the contractile component during isometric contractions at a given length [11]. The greater contractile component shortening would result in semitendinosus being at an even less advantageous position along its force–length curve at high angles of knee flexion. Neural inhibition may also be present across all hamstring muscles, and assessing motor unit function [31, 57] and the semitendinosus tendon reflex [93] may be beneficial for revealing any neural changes to the hamstrings after recovery from distal semitendinosus tendon harvesting. As such, there are likely multiple compounding factors leading to knee flexion weakness after semitendinosus tendon ACLR.

### Potential Synergist Compensation and Clinical Implications

Knee flexor synergists could not physiologically compensate for the post-ACLR loss of semitendinosus contribution to knee flexion moment at knee flexion angles where a between-leg difference was found experimentally. Indeed, an unrealistic F_max_ increase of 20% for all synergists was needed at 60°, and such a large increase was not even sufficient enough to compensate for the post-ACLR knee flexion weakness at 90°. Weakness being more prominent, and not compensatable, at 60° and 90° of knee flexion is well explained by the fact synergist muscles have their own unique functional roles [40]. The large semitendinosus moment arm in high degrees of knee flexion [6, 26] combined with the wide operating range of semitendinosus [12, 13, 63] results in semitendinosus being the greatest contributor to isometric maximal knee flexion moment at these joint angles (Fig. 1). Our simulations corroborate this angle specificity as semitendinosus atrophy had very little effect on overall flexion moment at 15° of knee flexion (Fig. 4), which may be expected as the other knee flexors are at or near their strongest in this knee position with the hip extended (Fig. 1). Therefore, tests of maximal isometric moment at low knee flexion angles or peak isokinetic moment, which occurs at these shallow knee angles [1, 16, 43, 60], are not specific for assessing impairments in semitendinosus function and may explain why studies have found no significant difference in peak isokinetic moment when comparing participants grouped by tendon regeneration status [17, 32].

Although the functional relevance of isometric weakness at 90° has been questioned [15], assessing strength at high angles of knee flexion is useful as it is more specific for semitendinosus. Relative to the other hamstring muscles, its long muscle fibers make semitendinosus better designed for high-velocity actions [89], such as sprint running [24, 80], that are performed faster than generally tested during isokinetic dynamometry assessment: maximal knee angular velocity during sprint running surpasses 800°/s [20, 47]. Indeed, semitendinosus metabolic activity is much greater than the other hamstring muscles when measured after repeated sprint running [5, 96], Therefore, the role of semitendinosus is not to just produce moment at high angles of knee flexion, but its large contribution to moment at these knee angles (Fig. 1) provides the best position for simple clinical assessment of its function (i.e., “manual muscle test” at 90° of knee flexion) that is otherwise masked by other traditional dynamometer tests and is quicker and more accessible than assessing T_2_ relaxation times from functional MRI. Further, measures at more extended knee joint angles may be obscured by substantial quadriceps atrophy [32, 74, 94], which results in a lower knee extension antagonist moment. This effect of quadriceps atrophy can outweigh potential effects of semitendinosus atrophy in certain conditions and is evident from the simulated increases in resultant knee flexion moment at these joint angles despite, for example, a reduced semitendinosus ACSA_max_ (Fig. 4). Overall, strength testing at 60° and 90° of knee flexion to specifically evaluate semitendinosus function should not be overlooked in clinical assessments as semitendinosus is designed differently than, and cannot be compensated for by, the other hamstrings, semitendinosus appears essential for athletic tasks such as sprint running [24, 80], and compromised semitendinosus function may increase the risk of muscle strain injury in other hamstring muscles [70].

The decrease in radial semitendinosus size only minimally contributed to the between-leg difference in strength, and the knee flexor synergists could not physiologically compensate for the impaired semitendinosus at 60° and 90°. Thus, while important, rehabilitation exercises aimed at inducing muscle hypertrophy are unlikely to lead to a full recovery of knee flexion moment, especially at angles of 60° or higher. Although muscle hypertrophy can be targeted during and after rehabilitation, substantially changing muscle length is practically impossible non-invasively once the tendon has regenerated and reattached. Using a harvesting technique that preserved the muscle-tendon junction and distal insertion of semitendinosus by harvesting only a partial width of the semitendinosus tendon, Sasahara et al. [69] found significantly less muscle retraction compared to harvesting the whole semitendinosus tendon, while the strength deficit at 90° of knee flexion was also substantially attenuated. Surgical technique aside, their results demonstrated the importance of maintaining semitendinosus muscle length when the distal semitendinosus tendon is harvested, which should be considered for future treatments and interventions. Given the implications of semitendinosus muscle shortening, measuring the between-leg difference in the distal muscle-tendon junction position, a proxy of semitendinosus shortening that can be measured using ultrasonography [33, 34], may be a beneficial assessment used in clinical practice.

### Limitations

The small number of non-regenerated tendon participants (*n* = 3) did not allow for statistical comparisons across subgroups, although comparing strength based on tendon regeneration status and the more complex modeling necessary for non-regenerated participants (e.g., accounting for potential myofascial force transmission) were not the main aims of our study. In our model, we set activation to maximal for all agonist muscles across all angles as the knee flexors demonstrate high-to-maximal levels of myoelectric activity across the range of motion [49, 61]. Therefore, assuming maximal activation allowed us to eliminate neural factors from the model and focus solely on the effects of morphological changes. As such, we also did not include the gastrocnemii in our model because the gastrocnemii EMG–joint angle curve during knee flexion MVC is unclear and fully activating the gastrocnemii is likely to overstate their contribution to isometric knee flexion (Supplementary Figure 3). Despite these limitations, simulated moments from our reference model were within one standard deviation of experimental values, demonstrating reasonable validity of the model [27]. Further, had we incorporated the gastrocnemii into our model, the relative contribution of semitendinosus to overall knee flexion moment would have been reduced at all joint angles. However, moment measures obtained at low angles of knee flexion already lacked sensitivity toward semitendinosus function. Additionally, a potentially lower relative contribution of semitendinosus at 60° and 90° of knee flexion would have resulted in an even greater mismatch between experimental measures and simulated effects of semitendinosus morphological changes. Therefore, neglecting the gastrocnemii was unlikely to influence our main conclusions. Regarding semitendinosus in the model, we assumed muscle fibers remained oriented in-parallel with the tendon after ACLR. Changes in fiber orientation may be possible given the different shape of semitendinosus after distal tendon harvest [51], although pennation appears to play a minor role in accurately modeling muscle force [71, 97]. We also did not account for any potential changes in semitendinosus moment arm that may occur depending on the new insertion site of the regenerated tendon. However, substantial weakness in 90° of knee flexion has been reported even when the insertion point of the regenerated hamstring tendons did not differ compared to the contralateral side [2] and any potential change in semitendinosus moment arm has been speculated to affect tibial internal rotation strength more than knee flexion moment [32]. Further, we only performed experiments with the hip in neutral position, the most common hip positioning in the literature for assessing knee flexion strength after ACLR [2, 16, 39, 43, 50, 55, 56, 69, 79, 83]. Nonetheless, knee flexion moment at 90° is also impaired in the seated position, albeit to a lesser extent compared to prone [79, 83], and using the prone position allowed us to reduce the additional effects of muscle lengthening via the hip. Finally, we investigated the effects of knee flexor morphology only on isometric knee flexion. Additional simulation studies will be beneficial for further elucidating functional effects of semitendinosus atrophy and any potential associations of knee flexion weakness with impaired semitendinosus function during dynamic tasks.

## Conclusion

Although the semitendinosus muscle is substantially smaller radially and longitudinally after distal tendon harvesting for ACLR, according to the modeling approach employed in this study, this biaxial atrophy does not fully explain the strength deficit during isometric MVC at high angles of knee flexion. Further, this strength deficit could not be completely compensated for by increasing the maximal force-generating capacity of synergist muscles within physiologically feasible limits. Overall, our results (i) suggest multiple interacting factors likely contribute to knee flexion weakness after distal semitendinosus tendon harvest for ACLR, even when the tendon regenerates, (ii) demonstrate evaluating isometric knee flexion strength at high angles of knee flexion provides a simple clinical test for assessing semitendinosus function, and (iii) highlight the need for considering surgical techniques and post-operative interventions that could preserve semitendinosus muscle length.

### Supplementary Information

Below is the link to the electronic supplementary material.

Supplementary file1 (TIF 11870 KB) Model estimates of soleus (top; black) and tibialis anterior (bottom; red) force using different active Gaussian shape factors. Superimposed are experimental muscle force-joint angle data from isometric maximal voluntary contractions extracted from Table 1 of Maganaris (2001). Model estimates were normalized to the muscle maximal isometric force, and experimental data were normalized to the highest value recorded in Table 1 of Maganaris (2001). The shape factor of 0.20 provided the most consistent approximation for the two muscles and was thus chosen to be representative of skeletal muscle for our study. Note that a shape factor of 0.45 was also plotted as this value was suggested by Thelen (2003)

Supplementary file2 (TIF 21548 KB) Model simulations of the effects of alterations of reduced maximal anatomical cross-sectional area (ACSA_max_; blue) and sarcomere number (red) and length (orange) at four knee joint angles with the hip in 90° of flexion. Reduced ACSA_max_ was also combined with fewer (purple) and shorter (green) sarcomeres. The model values are presented as medians, with the error bars representing the minimal to maximal values of the iterations used for simulations. Note no experimental data were available for comparison with these simulation

Supplementary file3 (TIF 11870 KB) Active fiber force (top) and knee flexion moment (bottom) for all knee flexor muscles, including the gastrocnemii, from full knee extension (0°) to 90° of knee flexion with the hip neutral (0°). Long (BFLH; red) and short (BFSH; orange) heads of the biceps femoris, gracilis (GRAC; blue), sartorius (SART; magenta), semimembranosus (SM; green), semitendinosus (ST; black), and medial (GAS MED; broken black) and lateral (GAS LAT; dash-dotted black) gastrocnemius muscles. Compare with Figure 1 of the main text. Note the likely unrealistically high knee flexion force and moment generated by the gastrocnemii under maximal activation. For this reason, the gastrocnemii were excluded from the model
