# Supplementary figures and images for: Muscle Morphology Does Not Solely Determine Knee Flexion Weakness After Anterior Cruciate Ligament Reconstruction with a Semitendinosus Tendon Graft: A Combined Experimental and Computational Modeling Study
Source: Ann Biomed Eng. 2024 Feb 29;52(5):1313–25. doi: 10.1007/s10439-024-03455-7 (PMC10995045; doi:10.1007/s10439-024-03455-7)

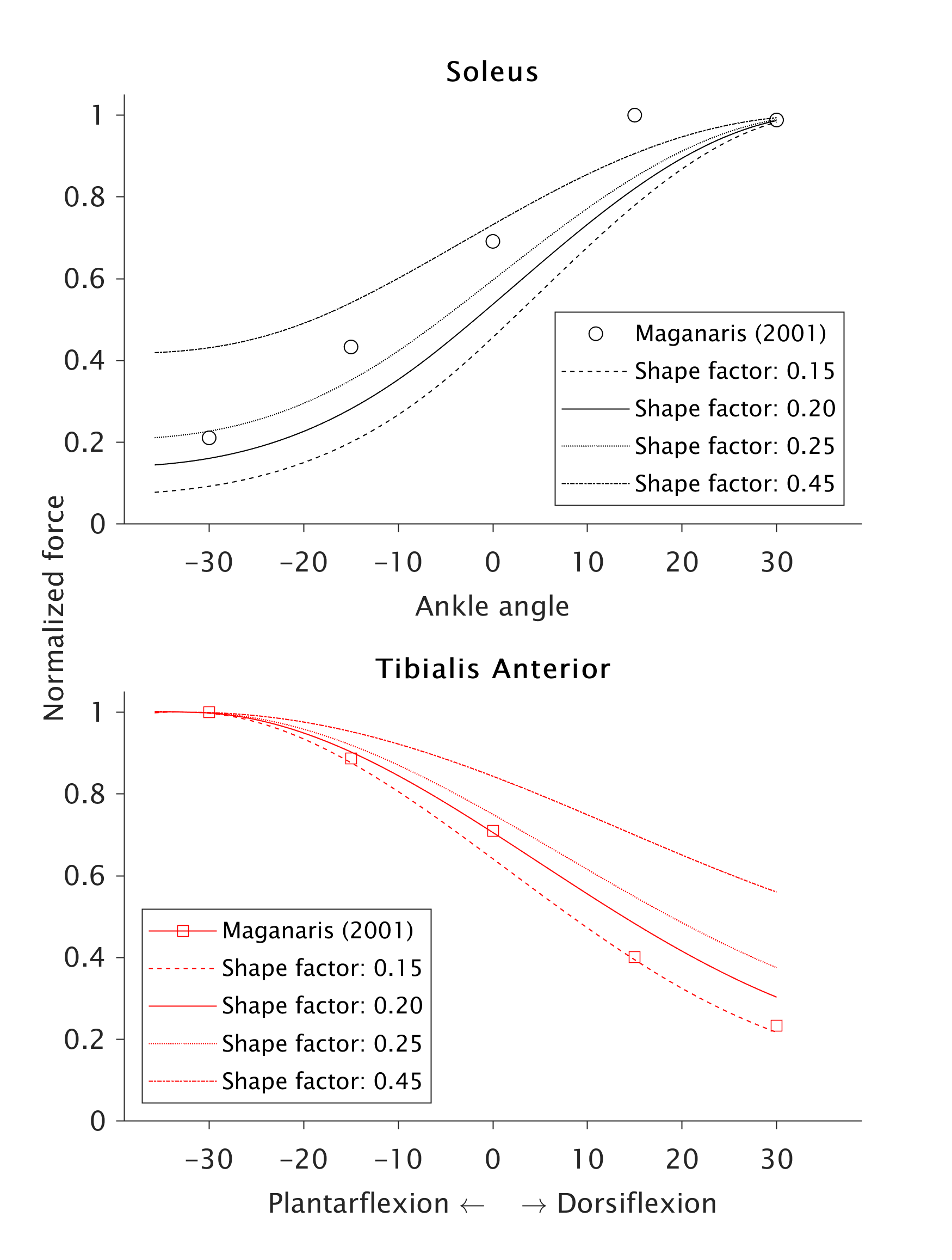

Supplement: Supplementary file 1 — Supplementary file1 (TIF 11870 KB) Model estimates of soleus (top; black) and tibialis anterior (bottom; red) force using different active Gaussian shape factors. Superimposed are experimental muscle force-joint angle data from isometric maximal voluntary contractions extracted from Table 1 of Maganaris (2001). Model estimates were normalized to the muscle maximal isometric force, and experimental data were normalized to the highest value recorded in Table 1 of Maganaris (2001). The shape factor of 0.20 provided the most consistent approximation for the two muscles and was thus chosen to be representative of skeletal muscle for our study. Note that a shape factor of 0.45 was also plotted as this value was suggested by Thelen (2003) [file 10439_2024_3455_MOESM1_ESM.tif]

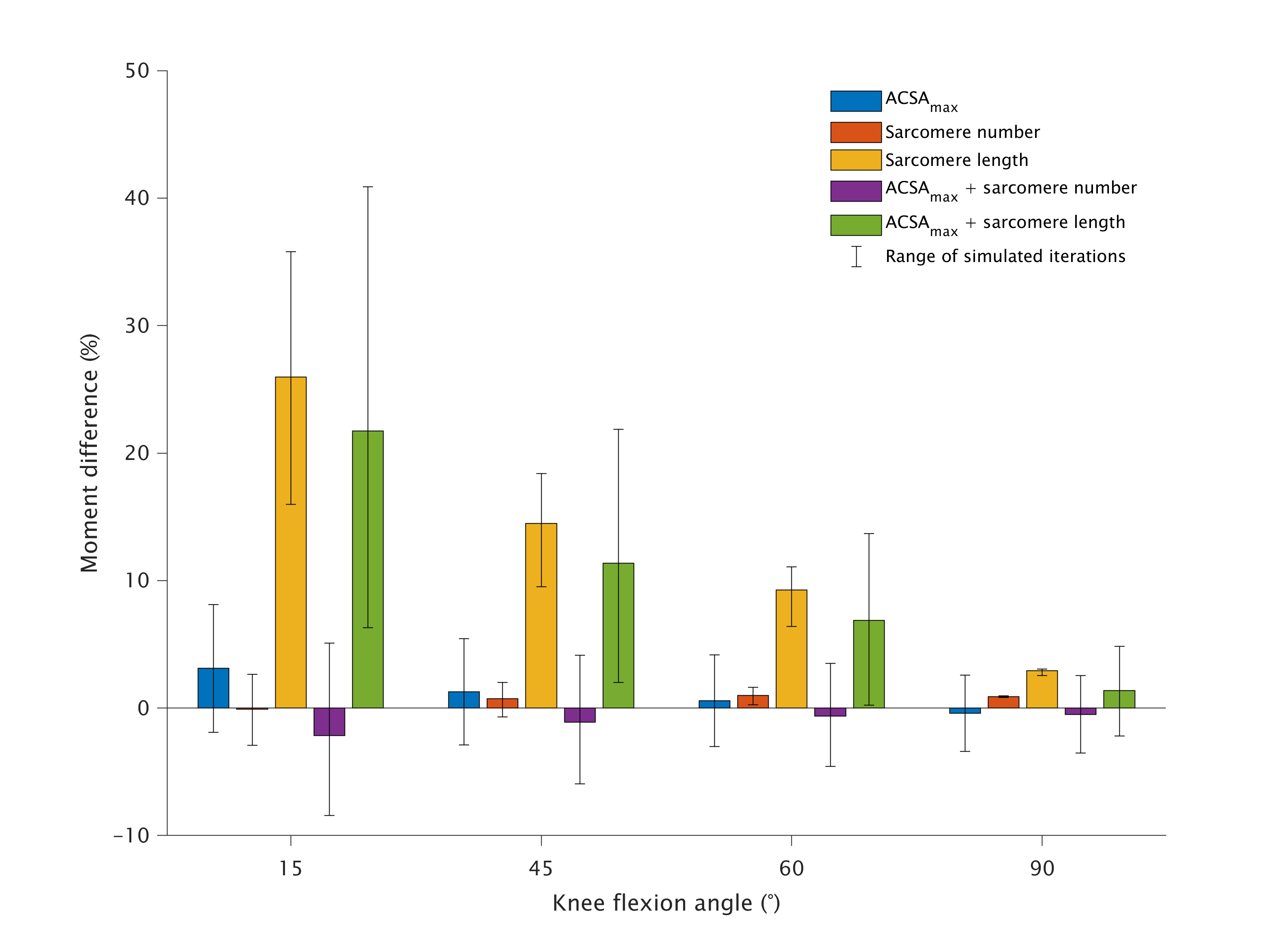

Supplement: Supplementary file 2 — Supplementary file2 (TIF 21548 KB) Model simulations of the effects of alterations of reduced maximal anatomical cross-sectional area (ACSAmax; blue) and sarcomere number (red) and length (orange) at four knee joint angles with the hip in 90° of flexion. Reduced ACSAmax was also combined with fewer (purple) and shorter (green) sarcomeres. The model values are presented as medians, with the error bars representing the minimal to maximal values of the iterations used for simulations. Note no experimental data were available for comparison with these simulation [file 10439_2024_3455_MOESM2_ESM.tif]

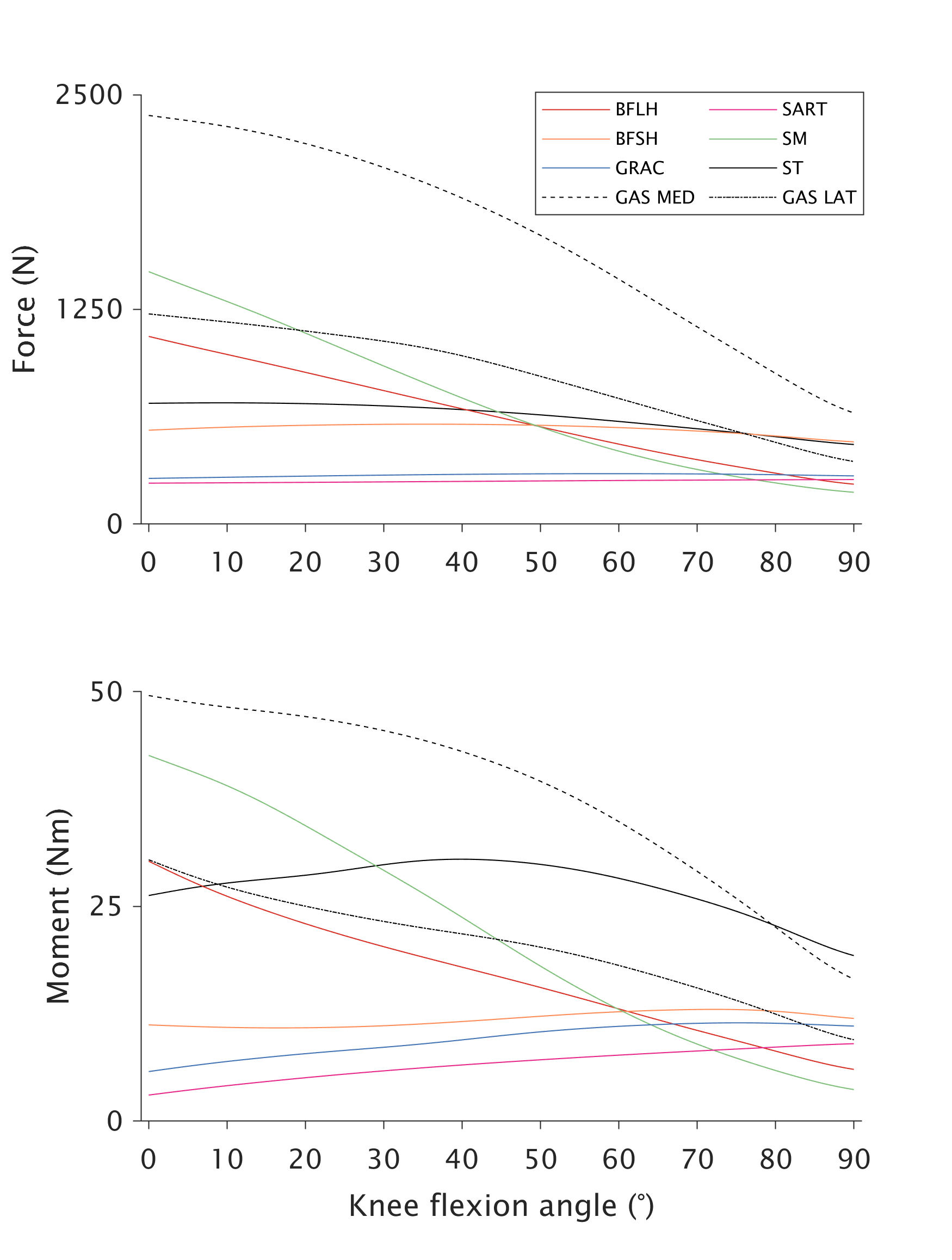

Supplement: Supplementary file 3 — Supplementary file3 (TIF 11870 KB) Active fiber force (top) and knee flexion moment (bottom) for all knee flexor muscles, including the gastrocnemii, from full knee extension (0°) to 90° of knee flexion with the hip neutral (0°). Long (BFLH; red) and short (BFSH; orange) heads of the biceps femoris, gracilis (GRAC; blue), sartorius (SART; magenta), semimembranosus (SM; green), semitendinosus (ST; black), and medial (GAS MED; broken black) and lateral (GAS LAT; dash-dotted black) gastrocnemius muscles. Compare with Figure 1 of the main text. Note the likely unrealistically high knee flexion force and moment generated by the gastrocnemii under maximal activation. For this reason, the gastrocnemii were excluded from the model [file 10439_2024_3455_MOESM3_ESM.tif]
